# Improved identifiability of myocardial material parameters by an energy-based cost function

**DOI:** 10.1007/s10237-016-0865-3

**Published:** 2017-02-10

**Authors:** Anastasia Nasopoulou, Anoop Shetty, Jack Lee, David Nordsletten, C. Aldo Rinaldi, Pablo Lamata, Steven Niederer

**Affiliations:** 1 Department of Biomedical Engineering, Division of Imaging Sciences and Biomedical Engineering, King’s College London, London, UK; 2 Cardiovascular Department, Guy’s and St. Thomas’ NHS Foundation Trust, London, UK

**Keywords:** Parameter estimation, Myocardium, Patient-specific modelling, Passive constitutive equations

## Abstract

Myocardial stiffness is a valuable clinical biomarker for the monitoring and stratification of heart failure (HF). Cardiac finite element models provide a biomechanical framework for the assessment of stiffness through the determination of the myocardial constitutive model parameters. The reported parameter intercorrelations in popular constitutive relations, however, obstruct the unique estimation of material parameters and limit the reliable translation of this stiffness metric to clinical practice. Focusing on the role of the cost function (CF) in parameter identifiability, we investigate the performance of a set of geometric indices (based on displacements, strains, cavity volume, wall thickness and apicobasal dimension of the ventricle) and a novel CF derived from energy conservation. Our results, with a commonly used transversely isotropic material model (proposed by Guccione et al.), demonstrate that a single geometry-based CF is unable to uniquely constrain the parameter space. The energy-based CF, conversely, isolates one of the parameters and in conjunction with one of the geometric metrics provides a unique estimation of the parameter set. This gives rise to a new methodology for estimating myocardial material parameters based on the combination of deformation and energetics analysis. The accuracy of the pipeline is demonstrated in silico, and its robustness in vivo, in a total of 8 clinical data sets (7 HF and one control). The mean identified parameters of the Guccione material law were $C_1=3000\pm 1700\,\hbox {Pa}$ and $\alpha =45\pm 25$ ($b_f=25\pm 14$, $b_{ft}=11\pm 6$, $b_{t}=9\pm 5$) for the HF cases and $C_1=1700\,\hbox {Pa}$ and $\alpha =15$ ($b_f=8$, $b_{ft}=4$, $b_{t}=3$) for the healthy case.

## Introduction

Left ventricular (LV) stiffness is proposed as a diagnostic indicator of cardiac function in heart failure (HF) patients (Westermann et al. 2008). Ventricular stiffness has been predominantly assessed in clinical practice through pressure–volume (p–V) analysis (Bermejo et al. 2013; Burkhoff et al. 2005; Zile et al. 2004). However, this approach is unable to distinguish between the anatomical and material contributions to LV stiffness. Specifically, an increment in ventricular size due to myocardial hypertrophy or an increase in collagen content with fibrosis may both lead to an equivalently stiffer LV behaviour using this methodology. Differentiating between these two components, anatomical and material, may improve the identification of HF aetiology in patients.

The development of biophysical models (Chen et al. 2016; Crozier et al. 2016; Krishnamurthy et al. 2013; Lee et al. 2015; Nordsletten et al. 2011; Plank et al. 2009) for the simulation of cardiac mechanics allows the distinct representation of the geometric and material components of stiffness. Using these models, the assessment of myocardial stiffness is posed as an inverse problem, where the material parameters are determined from known mechanical loads and deformations. Recent research in this field has focused on developing tractable pipelines for linking model parameters to data (Augenstein et al. 2005; Wang et al. 2009), evaluating the available material models (Criscione et al. 2002; Schmid et al. 2009) or improving the optimization strategies (Balaban et al. 2016; Moireau and Chapelle 2011; Moireau et al. 2008, 2009; Nair et al. 2007) and has led to the estimation of material parameters from clinical data sets (Asner et al. 2015; Gao et al. 2015; Wang et al. 2013; Xi et al. 2013). An inherent limitation in these current methods is the intercorrelation of the material parameters in myocardial material laws (Augenstein et al. 2006; Gao et al. 2015; Remme et al. 2004), which results in multiple parameter combinations corresponding to equivalent solutions in the optimization process. The existence of multiple solutions for the inverse problem limits the interpretation of these parameters for characterizing patient pathology and understanding changes in material properties under conditions of HF.

In this paper we investigate the role of the cost function (CF) in parameter identifiability and develop a novel energy-based CF that allows us to uniquely constrain the myocardial material parameters. For our analysis we choose a popular material model in cardiac mechanics, the transversely isotropic constitutive equation proposed by Guccione et al. (1991), which is reported to suffer from parameter coupling (Augenstein et al. 2006; Xi et al. 2011). We examine how a group of CFs based on geometric attributes, and the energy-based CF, constrain the optimization in the search of the parameters that best explain the clinical data of pressure and deformation. After an evaluation on a synthetic data set, a novel parameter estimation pipeline emerges based on the combined use of the energy-based CF with one of the geometric CFs, and is tested in 8 clinical cases demonstrating its ability to identify unique material parameters from patient data.

## Methods

In this section we summarize the synthetic and clinical data sets used (Sect. 2.1), the modelling framework (Sect. 2.2), the evaluated CFs (Sect. 2.3) and the proposed parameter estimation pipeline (Sect. 2.4). All data processing has been performed in *MATLAB*, and the meshes and simulation outputs have been visualized with *cmGui* 1 (Christie et al. 2002).

### Data sets

#### Synthetic

**Fig. 1:**
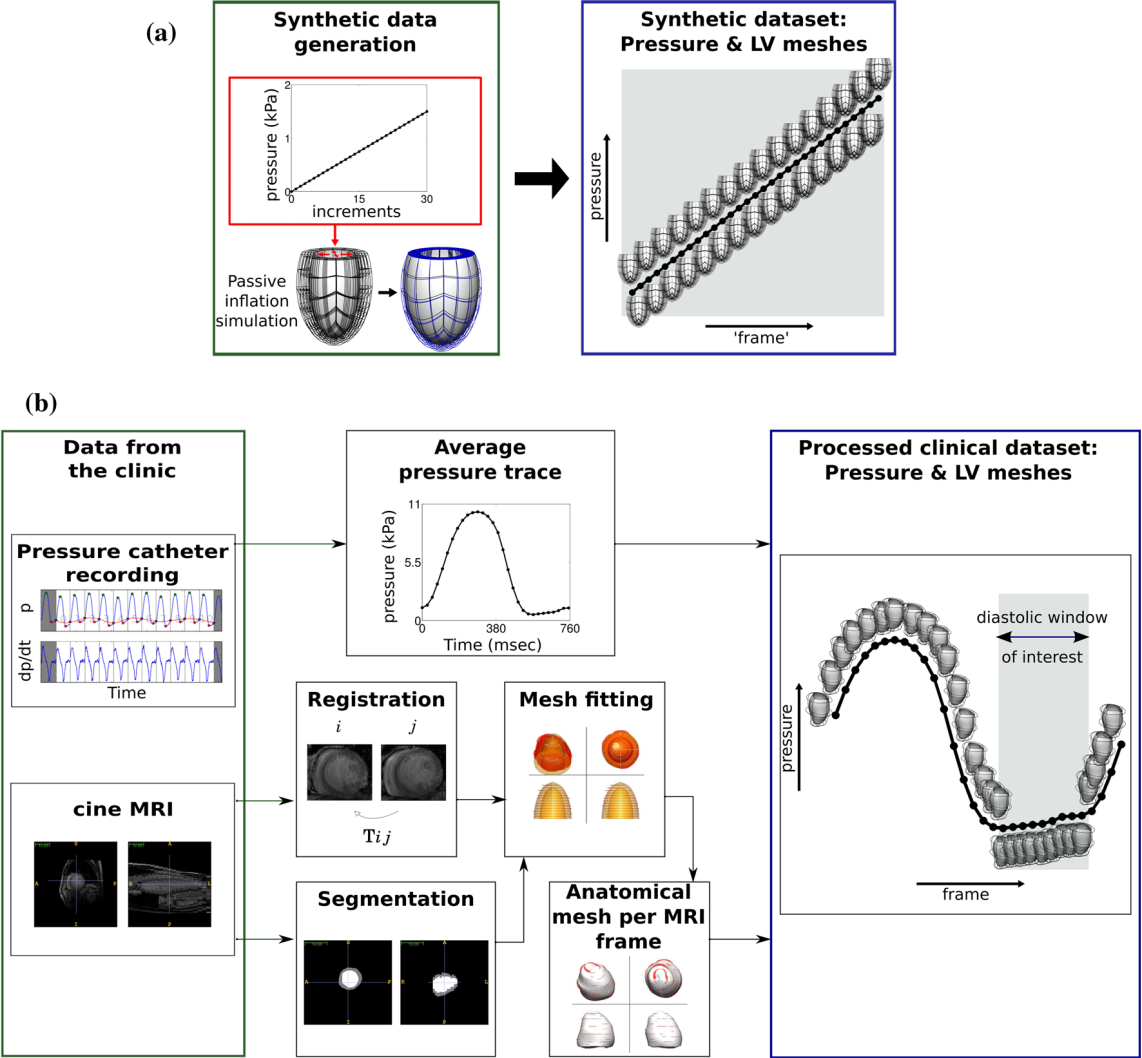
Synthetic and clinical data sets used to evaluate CFs. **a** Synthetic data set was created by applying 30 equal pressure increments to an idealized finite element (FE) model to generate 30 deformed geometries ‘frames’, **b** the clinical data set combines an averaged pressure trace with FE meshes that capture the deformation calculated from registration of the cine MRI frames. Combined, these provide a displacement and pressure measurement for each MRI frame recorded over the cardiac cycle. In our analysis only the diastolic frames, where a passive inflation approximation is relevant, are utilized (marked as diastolic window of interest)

To provide a known ground truth for the material parameters a synthetic data set was employed (see top panel in Fig. 1). An in silico model of the LV diastolic mechanics was created from the passive inflation of a truncated confocal prolate spheroidal of typical human cardiac dimensions representing the myocardial domain (Evangelista et al. 2011; Ho 2009; Humphrey 2002) to an end-diastolic pressure of 1.5 kPa (Humphrey 2002). A mesh of 320 (4 transmural, 16 circumferential, 4 longitudinal and 16 in the apical cap) hexahedral elements and 9685 nodes was used for the passive inflation simulation (details on the interpolation schemes and solver used are provided in Sect. 2.2.4). The pressure was applied over 30 equal pressure increments of 0.05 kPa, keeping the nodes of the ‘basal’ plane fixed in all directions. The resulting 31 meshes (undeformed mesh and 30 deformed meshes from each pressure increment) and their corresponding cavity pressure values from the simulation compose the synthetic data set used for the in silico study.

#### Clinical

In this study 8 clinical data sets are utilized, obtained from 7 Cardiac Resynchronization Therapy (CRT) patients (denoted as PC1-PC7) and one healthy control (denoted as HC). The clinical profile of the 8 cases is shown in Table 1. PC1-PC7 were obtained according to the clinical protocols followed in St Thomas’ Hospital, London, and consist of LV cavity pressure recordings and cardiac images covering the entire cardiac cycle. The data collection conforms to the principles of the Declaration of Helsinki and is guided by a local ethics committee approved protocol with patient informed consent. The healthy data set consists of pressure data and LV meshes covering diastole and were described previously (Xi et al. 2013).

The cardiac images of the CRT patient data sets (PC1–PC7) consist of 2-D short axis stacks of cine MRI with SENSE encoding ($1.19 \times 1.19 \times 8~\hbox {mm}^3$ to $1.45 \times 1.45 \times 10~\hbox {mm}^3$ resolution), taken on a 1.5T—in six out of seven cases—or 3T—in one case—Achieva Philips Medical Systems MRI scanner. Each MRI sequence had 25 to 35 frames with a temporal resolution between 23 and 32 msec. The LV domain was manually segmented in *itksnap* 2 from the end-diastolic frame. Images were processed using a non-rigid registration (Shi et al. 2013) which enables a spatially and temporally continuous description of the cardiac motion. Mesh personalization was performed on the segmented LV, as described previously (Lamata et al. 2014). A set of deforming finite element (FE) meshes, consisting of 12 to 16 (4 circumferential, 3 to 4 longitudinal and 1 transmural) cubic hexahedral elements and 436 to 580 nodes, were created for each patient by warping the personalized end-diastolic anatomical mesh using the motion field corresponding to each frame of the cine MRI. As a result, correspondence of material points between frames is obtained from the cine MRI images through the image registration and mesh personalization processes.

The LV cavity pressure transient was recorded during a catheterization procedure before the beginning of the CRT pacing protocols and separately from the MRI scans. For each patient an average pressure trace was calculated over 5–13 beats and then synchronized to the cavity volume trace estimated from the personalized FE meshes. The pressure–volume synchronization was based on the assumption that the inflection point in the pressure wave is approximately aligned with the R wave (acquisition time of the first frame of each MRI sequence) and finding the temporal offset that maximized the p–V loop area and was less than 5% of the R-R interval. The pressure transient was subsequently offset to ensure a zero pressure at the MRI phase that corresponded to the approximated reference configuration for the finite elasticity analysis, described below. The schematic of the steps followed for the processing of the clinical data sets is shown in the lower panel of Fig. 1.

### Mechanical model

LV diastolic passive inflation is simulated using large deformation mechanics assuming that deformation is driven principally by the LV pressure, the myocardium has homogeneous material properties, is incompressible, inertia or viscoelastic effects are negligible, that the LV is in a stable relaxed state in late diastole and that the right ventricle (RV), atria, pericardium and other neighbouring structures have secondary roles.

#### Cardiac microstructure

The myocardial microarchitecture requires the continuous description of the local fibre (*f*), sheet (*s*) and sheet normal (*n*) directions. In the model, local tissue microstructure was described by assuming a linearly varying preferential myocyte orientation from $-60^{\circ }$ at the epicardium to $60^{\circ }$ at the endocardium based on the findings by Streeter et al. (1969).

**Table 1:** Summary of the patient cases (PC1-PC7) and healthy data set (HC) used

Case	Age	Sex	EF (%)	ESV (ml)	EDV (ml)	EDP (kPa)
PC1	61	M	13.5	266	307	2.59
PC2	61	M	6.2	348	371	1.21
PC3	70	M	19.5	174	216	4.44
PC4	76	F	32.3	86	127	1.84
PC5	57	F	19.3	214	265	2.99
PC6	65	M	29.7	122	173	1.17
PC7	39	M	19.7	176	219	2.98
HC	36	M	$-^{\mathrm{a}}$	$-^{\mathrm{a}}$	134	1.89

$^{\mathrm{a}}$ Data not available

The abbreviations used are as follows: *EF* ejection fraction, *ESV* end-systolic volume, *EDV* end-diastolic volume all corresponding to the LV

Volumes were estimated from the personalized meshes following the p–V synchronization

#### Material description

The myocardium is modelled as a hyperelastic incompressible transversely isotropic material with the constitutive relation introduced by Guccione et al. (1991). The mathematical description of the Guccione law is given in Eqs. 1 and 2. The parameters $b_f$, $b_t$, $b_{ft}$ assign different mechanical responses to the tissue along the fibre (*f*) direction, across the transverse planes (*t*) and in the fibre-transverse shear planes (*ft*), respectively. The *f*,*s*,*n* indices in the strain components express projections of the Green–Lagrange strain tensor ($\varvec{E}$) along the fibre (*f*), sheet (*s*) and sheet normal (*n*) directions.

1 $$\begin{aligned} \varPsi= & {} \frac{1}{2} C_1 (\mathrm {e}^Q-1) \end{aligned}$$

2 $$\begin{aligned} Q= & {} b_{f} {E_{ff}}^2 + b_{ft} (2 {E_{fs}}^2 + 2 {E_{fn}}^2 )\nonumber \\&+\, b_{t} ( {E_{ss}}^2 + {E_{nn}}^2 + 2 {E_{sn}}^2 ) \end{aligned}$$

After the reformulation proposed by Xi et al. (2013), the strain energy density ($\varPsi$) is expressed as a function of the scaling ($C_1$) and bulk exponential ($\alpha$) parameters, along which the primary coupling occurs (Eqs. 1, 3).

3 $$\begin{aligned} Q= & {} \alpha [r_{f} {E_{ff}}^2 + r_{ft} (2 {E_{fs}}^2 + 2 {E_{fn}}^2 )\nonumber \\&+\, r_{t} ( {E_{ss}}^2 + {E_{nn}}^2 + 2 {E_{sn}}^2 )] \end{aligned}$$

4 $$\begin{aligned} \alpha= & {} b_{f}+b_{ft}+b_{t} \end{aligned}$$

5 $$\begin{aligned} r_{f}= & {} b_{f}/\alpha \ , \ r_{ft}=b_{ft}/\alpha \ , \ r_{t}=b_{t}/\alpha \end{aligned}$$

6 $$\begin{aligned}&r_{f}+ r_{ft}+ r_{t}=1 \end{aligned}$$

The $r_{f}, r_{ft}, r_{t}$ parameters are referred to as anisotropy ratios and range between 0 and 1, with the $r_f$ ratio obtaining usually the highest value in order to represent a stiffer behaviour along the fibre direction. Throughout our analysis the anisotropy ratios were kept constant at $r_{f}=0.55, r_{ft}=0.25, r_{t}=0.2$ while focusing on fitting the coupled $C_1$ and $\alpha$ parameters.

**Fig. 2:**
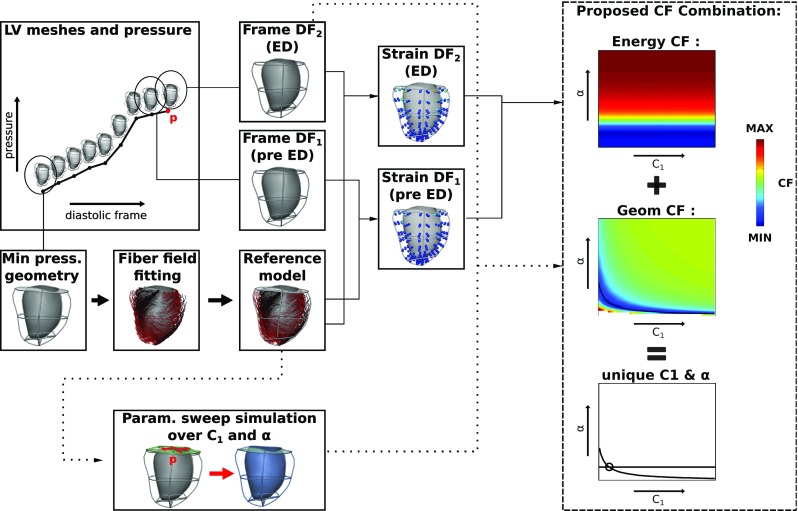
Overview of the proposed parameter estimation pipeline. The required input consists of LV meshes and corresponding pressure values at the defined diastolic window of interest (covering the frames from minimum pressure until before the beginning of contraction, see also Fig. 1). The evaluation of the energy-based CF is entirely data based, while the evaluation of the geometry-based CF requires the performance of mechanical simulations with sweeps over $C_1$ and $\alpha$. The combination of the CFs ensures the unique estimation of these parameters

#### Reference configuration

The reference configuration represents an idealistic stress- and strain-free geometry for the myocardium which is never reached within the cardiac cycle. For simplicity the LV geometry associated with the MRI frame corresponding to the minimum pressure was chosen as an approximation of the reference geometry.

#### Mechanical simulations and boundary conditions

The evaluation of the geometry-based CFs involves the performance of mechanical simulations where the LV was passively inflated to end-diastolic pressure applied on the endocardial surface of the LV mesh. The motion of the basal plane nodes was prescribed from the data, which for the case of the synthetic data set translates to maintaining a fully fixed basal plane. All boundary conditions (BCs) were applied in 30 equal increments. Figure 2 schematically shows where BCs are applied and how they are determined from the clinical data.

The finite elasticity problem was solved within a multifield variational principle approach, with incompressibility enforced through a Lagrange multiplier. Cubic and linear Lagrange interpolation were chosen for the displacement field and pressure, respectively (Hadjicharalambous et al. 2014). The mechanical simulations were performed in the *CHeart* 3 nonlinear FE solver following a Galerkin FE method (Lee et al. 2016).

### Examined CFs and their evaluation

#### Methodology to assess CF performance

To assess the parameter identifiability provided by the geometric and energy-based CFs, we computed the CF residual across the $\alpha$-$C_1$ parameter space. We then visualize the landscapes of the CF residuals, and locate the parameter subspaces that could potentially be identified as solutions to the inverse problem. This, for the case of the geometry-based CFs, requires the performance of mechanical simulations with parameter sweeps over $C_1$ and $\alpha$. Conversely, the energy-based CF relies only on data analysis, as highlighted below.

#### Geometry-based CFs

We evaluate the ability of six geometrically based CFs to uniquely constrain the passive material parameters either independently or in combination. The geometry-based CFs are based on popular CFs from the literature, comparing displacement, strain and cavity volume (Asner et al. 2015; Gao et al. 2015; Hadjicharalambous et al. 2015; Mojsejenko et al. 2015; Xi et al. 2013), and extended to include widely used clinical indices, such as wall thickening and apicobasal deformation. The required data for all these CFs can be readily provided from available imaging modalities. For the CF evaluation we consider the time at end diastole (ED), where the largest amount of deformation can be observed in the diastolic window. Specifically, the examined geometry-based CFs are:

- $L^2$ **displacement norm**. The $L^2$ displacement norm CF is estimated by comparing the displacements between the simulated displacement ($u_{\mathrm{sim}}$) and the clinically measured or synthetic data ($u_{\mathrm{dat}}$):
  7 $$\begin{aligned} |\Delta \varvec{u}|=\sqrt{\frac{\int _\varOmega {({\varvec{u}}_{\mathrm{sim}}-{\varvec{u}}_{\mathrm{dat}})\cdot ({\varvec{u}}_{\mathrm{sim}}-{\varvec{u}}_{\mathrm{dat}}) \, \mathrm {d}\Omega }}{\int _\varOmega \mathrm {d}\Omega }} \, . \end{aligned}$$
- $L^2$ **strain norm**. The $L^2$ norm of the difference in Green–Lagrange strains between simulated (${\varvec{E}}_{\mathrm{sim}}$) and synthetic or clinical data (${\varvec{E}}_{dat}$):
  8 $$\begin{aligned} |\Delta \varvec{E}|=\sqrt{\frac{\int _\varOmega {({\varvec{E}}_{\mathrm{sim}}-{\varvec{E}}_{\mathrm{dat}}):({\varvec{E}}_{\mathrm{sim}}-{\varvec{E}}_{\mathrm{dat}}) \, \mathrm {d}\Omega }}{\int _\varOmega \mathrm {d}\Omega }} \, . \end{aligned}$$
  The L2 displacement and L2 strain norm CFs were estimated using 4 Gauss points per element direction. Increasing the Gauss points to 5 per element direction led to a maximum 5 10$^{-8}$ mm error for $|\Delta \varvec{u}|$ and 2 10$^{-7}$ error for $|\Delta \varvec{E}|$, which is well within the expected magnitude of error due to data noise.
- **Cavity Volume**. The Cavity Volume CF ($|\Delta \mathscr {V}|$) describes the absolute difference between the LV cavity volumes in the clinical or synthetic data ($\mathscr {V}_{\mathrm{dat}}$) and model simulations ($\mathscr {V}_{\mathrm{sim}}$):
  9 $$\begin{aligned} |\Delta \mathscr {V}| = | \mathscr {V}_{\mathrm{sim}}-\mathscr {V}_{\mathrm{dat}}| \, . \end{aligned}$$
- **Wall Thickness**. The wall thickness metric $|\Delta d_{WT}|$ compares the average wall thickness at the equatorial nodes between the simulation ($d_{WT}^{\mathrm{sim}}$) and the data ($d_{WT}^{\mathrm{dat}}$):
  10 $$\begin{aligned} |\Delta d_{WT}| = \frac{\sum \nolimits _{n_1}^{n_n} |d_{WT}^{\mathrm{sim}}-d_{WT}^{\mathrm{dat}}|}{n_n} \, , \end{aligned}$$
  where $n_1,\ldots ,n_n$ are the node pairs at the equator used for the wall thickness measurements.
- **Apicobasal distance**. The endocardial $|\Delta d_{\mathrm{ABendo}}|$ (or epicardial $|\Delta d_{\mathrm{ABepi}}|$) apicobasal distance metrics estimate the average difference between the distance of the endocardial (or epicardial) basal nodes to the endocardial (or epicardial) apical node at the data $d_{ABendo}^{dat}$ (or $d_{\mathrm{ABepi}}^{\mathrm{dat}}$) and simulation $d_{\mathrm{ABendo}}^{\mathrm{sim}}$ (or $d_{\mathrm{ABepi}}^{\mathrm{sim}}$) meshes:
  11 $$\begin{aligned} |\Delta d_{\mathrm{ABendo}}|= & {} \frac{\sum \nolimits _{m_1}^{m_n} |d_{\mathrm{ABendo}}^{\mathrm{sim}}-d_{\mathrm{ABendo}}^{\mathrm{dat}}|}{m_n}, \end{aligned}$$
  12 $$\begin{aligned} |\Delta d_{\mathrm{ABepi}}|= & {} \frac{\sum \nolimits _{m_1}^{m_n} |d_{\mathrm{ABepi}}^{\mathrm{sim}}-d_{\mathrm{ABepi}}^{\mathrm{dat}}|}{m_n}, \end{aligned}$$
  where $m_1,\ldots ,m_n$ are the basal nodes whose distance from the apex is calculated.

#### Energy-based CF

Based on the modelling assumptions described in Sect. 2.2, the energy conservation dictates the equality of the external work $W_{\mathrm{ext}}$ (the work performed by the external loads acting on the tissue) to the internal energy $W_{\mathrm{int}}$ (the work of the internal stresses and strains), giving:

13 $$\begin{aligned} W_{\mathrm{ext}} = W_{\mathrm{int}}. \end{aligned}$$

The internal energy for hyperelastic materials can be expressed via the strain energy function $\varPsi$, which with the chosen constitutive law (Eqs. 1 and 3) yields the internal energy expression in Eq.  as a function of the Green–Lagrange strain tensor $\varvec{E}$.

14 $$\begin{aligned} W_{\mathrm{int}} = \int _V \varPsi dV = \int _V \frac{1}{2} C_1 (\mathrm {e}^{Q(\varvec{E})}-1) dV \end{aligned}$$

The external work is estimated as the increase in LV cavity volume ($\mathscr {V}$) from the reference configuration ($\mathscr {V}_{0}$) to a given volume $\mathscr {V}_{D}$ caused by the LV pressure *p*.

15 $$\begin{aligned} \begin{aligned} W_{{\mathrm {ext}}}&=\int _{\mathscr {V}_{0}}^{\mathscr {V}_{D}} p d\mathscr {V} \end{aligned} \end{aligned}$$

Defining two points in diastole with corresponding volume, Green–Lagrange strain, external work and internal energy of $\mathscr {V}_{1}$, $\varvec{E_1}$, $W_{\mathrm{ext}1}$ and $W_{\mathrm{int}1}$ and $\mathscr {V}_{2}$, $\varvec{E_2}$, $W_{\mathrm{ext}2}$ and $W_{\mathrm{int}2}$, respectively, we can write :

16 $$\begin{aligned} \frac{W_{\mathrm{ext}1}}{W_{\mathrm{ext}2}} = \frac{W_{\mathrm{int}1}}{W_{\mathrm{int}2}} \, . \end{aligned}$$

The ratios of the external work and internal energy at these two points must be equal and the difference of these two ratios should tend to zero. This provides the energy-based CF, *f*:

17 $$\begin{aligned} f = \frac{W_{\mathrm{ext}1}}{W_{\mathrm{ext}2}} - \frac{W_{\mathrm{int}1}}{W_{\mathrm{int}2}}. \end{aligned}$$

Substituting in the definition of $W_{\mathrm{int}}$ (Eq. 14) and $W_{\mathrm{ext}}$ (Eq. 15) then gives :

18 $$\begin{aligned} f = \frac{\int _{\mathscr {V}_{0}}^{\mathscr {V}_{1}} p d\mathscr {V}}{\int _{\mathscr {V}_{0}}^{\mathscr {V}_{2}} p d\mathscr {V}} - \frac{\int _V \frac{1}{2} (\mathrm {e}^{Q(\varvec{E}_{1})}-1) dV }{\int _V \frac{1}{2} (\mathrm {e}^{Q(\varvec{E}_{2})}-1) dV }. \end{aligned}$$

We can see in Eq. 18 that the constant $C_1$ of the constitutive Eq. 14 is cancelled out from the numerator and denominator of the right part of Eq. 17. Note that here the strain field is directly derived from the deformation field extracted from the medical images, without any forward simulation involving a choice of $C_1$, giving a CF dependent only on the material parameters in *Q*.

In implementing the energy CF we select two time points. In the clinical study these correspond to diastolic frames (*DF*) of the MRI sequence. To avoid potential artefacts from slow decaying active tension we choose to use frames from the MRI that corresponded to the last two frames of end diastole (this choice is reviewed in Appendix 7). We define $DF_2$ as the end-diastolic frame, and $DF_1$ as the frame prior to $DF_2$ (see also Fig. 2). For consistency, we also chose to use the last two ‘frames’ in the analysis of the synthetic dataset. These correspond to the last two increments of the simulation used to generate it ($DF_1$ corresponds to the solution after inflation to 1.45 kPa and $DF_2$ to 1.5 kPa ).

The external work for each time point is calculated by integrating the product of the pressure and change in volume between sequential MRI frames, giving:

19 $$\begin{aligned} W_{\mathrm{ext}}= \sum \limits _{n=RF}^{MRI_F -1} \frac{p_{n}+p_{n+1}}{2} (\mathscr {V}_{n+1}-\mathscr {V}_{n}). \end{aligned}$$

In Eq. 19, *RF* corresponds to the index of the cine sequence that corresponds to the reference frame, $MRI_F$ corresponds to the index of the MRI frame of interest (for example the index for $DF_1$ or $DF_2$), $p_{n}$ is the pressure at MRI frame *n* and $\mathscr {V}_{n}$ is the LV cavity volume at MRI frame *n*.

The internal energy $W_{\mathrm{int}}$ is calculated solely from the Green–Lagrange strain field which is derived from the displacement field between the geometries of the *DF* under consideration and the MRI frame employed as the reference frame. This tensor field can be calculated directly from the image registration algorithm without any further requirement for mechanical simulations of the LV model.

The energy-based CF is only dependent on the parameters in *Q* in the Guccione law. Assuming constant anisotropy ratios then allows the $\alpha$ parameter (Eq. 3) to be uniquely inferred form the energy-based CF.

### Proposed parameter estimation workflow

The proposed workflow relies on the combination of the energy-based and the $L^2$ displacement norm ($|\Delta \varvec{u}|$) CFs, inferring the unique $C_{1}-\alpha$ parameter set from the point of intersection between the minimum residual contours of the two CFs (Fig. 2). Thus, the steps are:

- *Step 1.* Estimate $\alpha$ through minimization of the energy-based CF from analysing the data.
- *Step 2.* Perform mechanical simulations in order to optimize $C_1$ from the $|\Delta \varvec{u}|$ CF.

The $L^2$ displacement norm is chosen as the geometric CF (Sect. 2.3.2) for the $C_1$ parameter estimation due to its robustness (see Sect. 3.2.1) and the comprehensive data model deformation comparison it provides compared to simpler metrics. Note that the optimization of $C_1$ can be achieved by setting $\alpha$ to the value obtained in step 1 and sweeping over $C_1$, resulting in 1D searches of the $C_1$ value that minimizes $|\Delta \varvec{u}|$. However, $C_1$ estimation through 1D optimization may not always be possible, as for certain $C_1$, $\alpha$ combinations the nonlinear mechanical solver may not converge (Land et al. 2015b). To overcome this, simulations with 2D sweeps over both $C_1$ and $\alpha$ can be performed in order to allow an exponential fit of the parameter combinations yielding the minimum $|\Delta \varvec{u}|$ residual (for a justification on the choice of the exponential fit, see Xi et al. (2013)). $C_1$ can then be uniquely estimated from the intersection of this curve with the flat line corresponding to the $\alpha$ solution from step 1 (see Fig. 5c for an example). The latter approach was followed in our study for parameter estimation from both synthetic and clinical datasets.

**Fig. 3:**
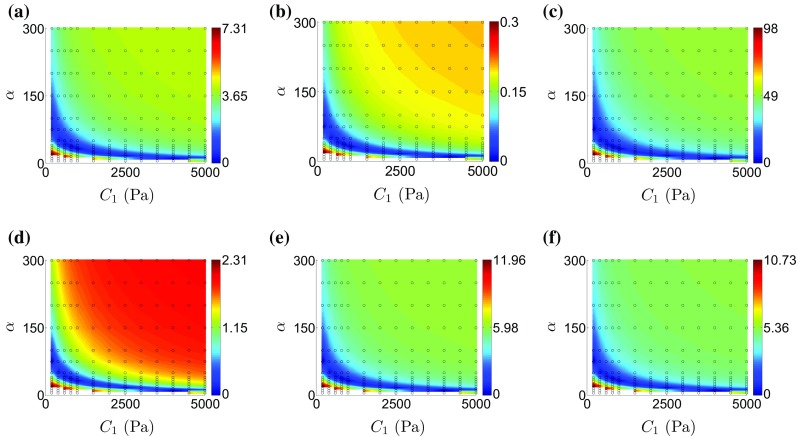
Plots of landscapes of the examined geometry-based CF residuals over the $C_1$ and $\alpha$ parameter space for the synthetic data set: **a** $|\Delta \varvec{u}|$ CF (residual in mm). **b** $|\Delta \varvec{E}|$ CF. **c** $|\Delta \mathscr {V}|$ CF (residual in ml). **d** $|\Delta d_{WT}|$ CF (residual in mm). **e** $|\Delta d_{\mathrm{ABendo}}|$ CF (residual in mm). **f** $|\Delta d_{\mathrm{ABepi}}|$ CF (residual in mm). The parameter grid used is shown as the empty *black circles* and is in the range of 200–5000 Pa for $C_1$ and 5–300 for $\alpha$. The parameter combinations yielding the minimum CF residual are plotted in blue. The white patches in the plots a–f signify parameter combinations that resulted in simulations that could not solve with the defined loading paradigm

## Results

### Parameter estimation in the synthetic data set

To determine if geometric CFs or the energy-based CF can constrain passive stiffness parameters, in the absence of data noise and under conditions of absolute model fidelity to the data, we evaluate the CF performance on a synthetic data set with baseline Guccione constitutive law parameters set to $\alpha =30$, $C_1=1000~\hbox {Pa}$, $r_f=0.55$, $r_{ft}=0.25$, $r_{t}=0.2$ (Eqs. 1, 3).

#### Identifiability of the geometry-based CFs

**Fig. 4:**
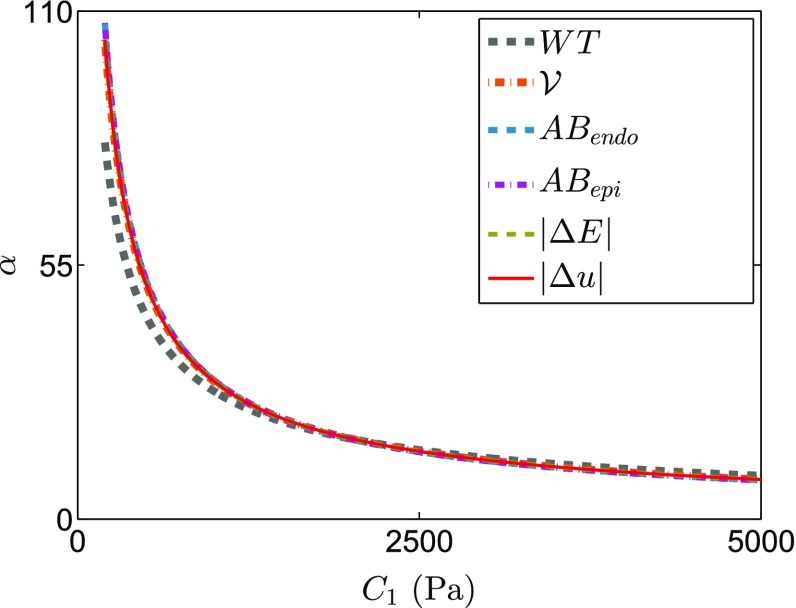
Lines of minimal residual for all the geometry-based CFs in the synthetic dataset, after an exponential fitting (each line is an exponential fitting to the points of minimum residual, see Fig. 5c for an example). The result shows that the lines of minimal residual are nearly identical for all geometry-based CFs in silico

The reported coupling between the $C_1$-$\alpha$ parameters (Xi et al. 2013) is confirmed for the $L^2$ displacement norm (Fig. 4a) and extended for the remaining geometry-based CFs (Fig. 4b–f). Fitting an inverse exponential function to the parameters with the minimum residual for each CF reveals that the CF minimization contours are highly coincident (Fig. 6). This shows that for the in silico case the geometry-based CFs, independently or in combination, are unable to uniquely constrain the parameters of the Guccione law.

#### Identifiability of the energy-based CF 

The landscape of the energy-based CF residual in the $C_1$-$\alpha$ parameter space is shown in Fig. 3. Due to its formulation the energy-based CF is independent of the $C_1$ parameter, as is evident by the fact that its minimization contour is parallel to the $C_1$ axis and the minimum occurs for a unique value of $\alpha$. Combining the energy-based CF with the $L^2$ displacement norm the ground truth $C_1$, $\alpha$ parameters of the synthetic dataset were recovered (Fig. 3).

### Parameter estimation in the clinical data sets

Following the in silico analysis we investigated the CF performance in 8 clinical cases.

#### Evaluating Geometric CFs on Clinical Data

**Fig. 5:**
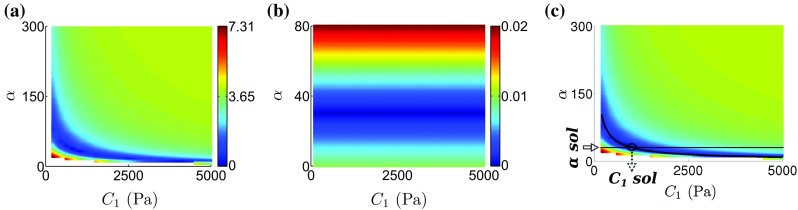
Proposed CF combination for unique parameter estimation emerging from the in silico analysis. **a** The landscape of the $|\Delta \varvec{u}|$ CF (residual in mm), which is the chosen geometric CF (see Sect. 2.4 for more details). **b** Landscape of the energy-based CF. A straight line pattern emerges that demonstrates the independence of the CF on $C_1$ and its ability to uniquely identify $\alpha$. **c** Unique parameter estimation with the combined use of the energy-based CF for $\alpha$ parameter identification and the $L^2$ displacement norm CF. A superimposed black curve is fitted to the parameter combinations corresponding to the minimum $|\Delta \varvec{u}|$ CF residual values. The horizontal black line corresponds to the minimum energy-based CF residual. The identified parameters (indicated by the black circle at the intersection of the energy-based and geometry-based CF minimization lines) coincide with the ground truth values ($\alpha =30$, $C_1=1000~\hbox {Pa}$). (Minimum and maximum CF residuals are shown in blue and red, respectively.)

The energy-based CF must be paired with a geometric CF to constrain both the $C_1$ and $\alpha$ parameters. To determine the geometric CF to pair with the energy-based CF we evaluated the six proposed geometric CFs on the 8 clinical data sets. The identifiable parameter combinations for each CF for each clinical data set are presented in Fig. 5 as summary plots of the exponential fits to the CF residual minimization parameter contours. This figure confirms that the $C_1$-$\alpha$ parameter coupling exists in vivo for all the geometric CFs. However, the minimization contours are not always coincident in the clinical setting, with some of the CF producing discordant parameter solutions as in cases PC2 and PC7 in Fig. 5.

The $L^2$ norm of displacements was selected as the geometric CF to pair with the energy CF, as it is based on a thorough global comparison of the agreement of the deformation field between model and data and consistently accorded well with the majority of the other geometric CFs across cases.

#### Identifiability of the energy-based CF 

The energy-based CF was estimated for the 8 clinical data sets. Its independence on $C_1$ is verified in clinical data, as the CF minimizing parameter combinations form a horizontal line parallel to the $C_1$ axis (see Fig. 5c, where the fitted line to the minimum residual contour is overlain on top of the exponential fits to the geometry-based CF minimums).

#### Estimated Parameters from the proposed pipeline

Following the proposed pipeline (Sect. 2.4), the passive material parameters for the 8 clinical data sets were determined from the intersection of the fits to the minimum residuals of the energy and $L^2$ displacement norm CFs (Fig. 5). The identified parameters are shown in Table 2 along with the $L^2$ displacement norm residual for each case.

**Table 2:** Parameter estimation results from the application of the proposed pipeline to the clinical data sets

**Case**	$\alpha _{\mathrm{sol}}$	$C_1$ (Pa)	$|\Delta \varvec{u}|$ (mm)
PC1	61	5300	0.95
PC2	61	820	1.96
PC3	66	1960	2.56
PC4	5	4780	5.61
PC5	24	3140	$-^{\mathrm{b}}$
PC6	29	1460	1.91
PC7	66	3300	1.18
HC	15	1700	3.35

$^{\mathrm{b}}$ In case PC5 there is no available residual as the forward simulation with the identified parameters did not converge.

The $\alpha$ parameter is estimated by the energy-based CF and the $C_1$ parameter from the $|\Delta \varvec{u}|$ CF

A certain level of variability is evident in the estimated parameters. In HF patient cases, $C_1$ ranges from 820 Pa for case PC2 to 5300 Pa for PC1, and $\alpha$ from 5 for PC4 to 66 for PC3 and PC7. The healthy volunteer data set yielded 1700 Pa for $C_1$ and 15 for $\alpha$. To provide context for the fitted parameters in the parameter estimation results of this study, previous estimates of the Guccione parameters fitted to human data are presented in Table 3.

**Table 3:** Estimated $C_1$, $\alpha$ parameters for human myocardium from previous studies

Case	$C_1$ (Pa)	$\alpha$	$|\Delta \varvec{u}|$ (mm)
Human			
Healthy$^{\mathrm{a}}$	$2000 ^{\mathrm{e}}$	43	1.78
Patient 1$^{\mathrm{a}}$	$2000 ^{\mathrm{e}}$	105	1.58
Patient 2$^{\mathrm{a}}$	$2000 ^{\mathrm{e}}$	95	1.39
Healthy$^{\mathrm{b}}$	$3600 \pm 1200^{\mathrm{e}}$	38	–
HT$^{\mathrm{b,c}}$	$12000 \pm 2600 ^{\mathrm{e}}$	38	–
NI-HF$^{\mathrm{b,d}}$	$11800 \pm 3400 ^{\mathrm{e}}$	38	–

$^{\mathrm{a}}$ Xi et al. (2013). $^{\mathrm{b}}$Wang et al. (2013). $^{\mathrm{c}}$ Patients with hypertrophic LV. $^{\mathrm{d}}$ Patients with non-ischaemic HF with reduced EF. $^{\mathrm{e}}$ In these studies the scaling constant is defined as half the $C_1$ parameter in Eq. 1, and therefore, values reported here have been doubled for consistency in the results

### Comparison with Previous Methods

We test if the proposed energy CF method for unique parameter estimation predicts significantly different local stresses as compared to one of the previous approaches, specifically where $C_1$ was fixed to 2000Pa (Xi et al. 2013). The difference in stresses developed at the ED frame using the two methods is presented in Table 4. The large discrepancies observed in some cases, notably PC2, can be explained by the existence of large local strains that amplify differences due to the exponential term in the strain energy function. In addition, Fig. 7 illustrates the difference in stress–strain curves corresponding to a 1-D fibre stretch for the parameter pairs estimated with both methods.

**Table 4:** Differences in stress calculated with parameters estimated by proposed method (*A*) and a previous one (Xi et al. 2013), where $C_1$ was fixed at 2000 Pa (*B*)

**Case**	Mean ${\hat{S}_{ff}}^A-{\hat{S}_{ff}}^B$ (Pa)	Standard deviation (Pa)	${\alpha }^B$
Synth	31.7	102.8	17
PC1	$-$461.8	47103.7	142
PC2	$-$577767.5	34820092.3	25
PC3	3127.3	52912.1	65
PC4	$-$7.3	284.9	10
PC5	$-$0.5	82.2	35
PC6	32.6	114.1	22
PC7	18.7	598	92
HC	45.7	972.7	13

Stress values are the deviatoric second Piola–Kirchhoff stress in the fibre direction (${\hat{S}_{ff}}$) computed at end diastole. Estimated $\alpha$ from fixing $C_1$ (${\alpha }^B$) are also reported for completeness

## Discussion

We have shown that unique identification of myocardial material parameters is possible with a suitable choice of the CF. To our knowledge, this is the first time that the two coupled parameters in the Guccione model have been uniquely constrained by clinical data; previously this issue was addressed by fixing part of the parameter set (Asner et al. 2015; Hadjicharalambous et al. 2015; Wang et al. 2013; Xi et al. 2013).

### Identifiability by an energy-based CF

The core methodological contribution of this work is the proposal of a CF that removes the parameter coupling. The energy-based CF identifies $\alpha$ due to its independence to the $C_1$ parameter. Its accuracy was tested in silico, where it estimates the correct $\alpha$ parameter and combined with the $L^2$ displacement norm provides accurate estimates of the ground truth parameter values. Results in 8 real clinical data sets with the complete pipeline demonstrate that the CF is robust to the inherent noise in clinical data and finite model fidelity.

The novel energy-based CF is also a data driven metric. Only the data of deformation (strain and cavity volume) and pressure are required to compute it, without the need of computational simulations or data assimilation pipelines. This has three main benefits. Firstly, the data derived deformation field employed in the CF is unaffected by the $C_1$-$\alpha$ coupling that arises from the simulation. Secondly, the computationally expensive search over the full parameter space involved in current data assimilation schemes has one dimension of the parameter space reduced since the $\alpha$ parameter is fixed. Thirdly, the reduction of methodological complexity to obtain the $\alpha$ parameter opens the possibility for a quicker and easier clinical adoption.

It is important to note that the energy-based CF raises the demands on data quality and quantity, since it requires strain data of the entire myocardium at two time points during diastole and the pressure–volume information covering the filling phase of the cycle. The importance of data quality on parameter estimation is demonstrated in a sensitivity study, provided in Appendix 6. In the absence of high fidelity strain data it is possible to recast the energy CF in terms of a pressure CF. This allows unique parameter estimates from pressure and volume transient data alone. The efficacy of this approach is presented in Appendix 8.

In our calculations the external work is estimated using a pressure–volume approach (see Eq. 19) which is fully accurate for the case of a deformation field consistent to the passive inflation assumption we have adopted. However, the image driven Dirichlet boundary conditions applied on the basal plane in the clinical data sets contribute to external work. This contribution is quantified as a mean 5% of the elastic energy in the clinical cases based on forward simulations with the identified parameters.

The efficiency of the energy-based CF was demonstrated for the Guccione material law, but can be extended to other exponential constitutive relations for reducing the material parameter redundancy by one, such as the Holzapfel–Ogden law (Holzapfel and Ogden 2009) as demonstrated in Appendix 9 or the pole–zero (Nash and Hunter 2000) among others.

### Geometry-based CFs

Geometry-based CFs, and their combination, were not able to identify unique myocardial material parameters, agreeing with previous reports (Augenstein et al. 2006; Xi et al. 2011).

**Fig. 6:**
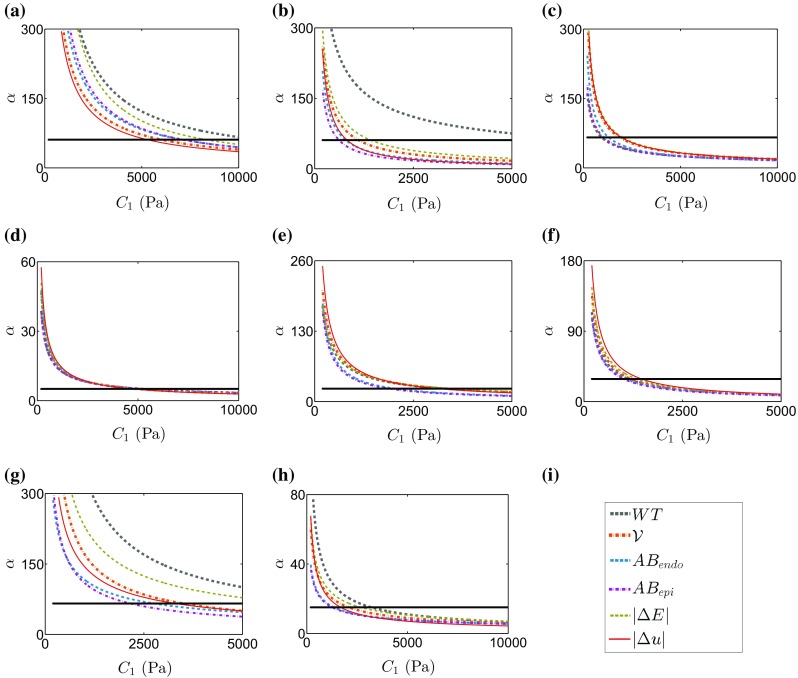
Lines of minimal residual from the geometry-based CF superimposed to the solution for the $\alpha$ parameter resulting from the energy-based CF (*flat line*) in each of the 8 cases studied: **a** PC1, **b** PC2, **c** PC3, **d** PC4, **e** PC5, **f** PC6, **g** PC7, **h** HC. **i** Legend

We investigated if a combination of geometrical CFs could improve parameter identifiability. The $C_1$- $\alpha$ parameters would then be identified by an intersection of the minimization contour of two or more CFs. Nevertheless, in the in silico data set the minimization contours are almost identical for the different CFs, suggesting the low complementary value of the CFs (Fig. 4). On the contrary, results with real data report a large variability in the agreement between the different CFs in half of the cases (see the offsets between lines that identify the coupling in Fig. 6), suggesting that this strategy does not lead to a unique set of parameters in practice. The disagreement between CFs with real data, and not with simulated data, is interpreted as a reflection of the mismatch between model and real data, caused by a combination of lack of model fidelity and data quality.

Note that while the geometric cost functions are based on a single frame, in contrast to the two frames used in the energy CF, the addition of an adjacent frame is not expected to improve the identifiability of parameters from geometric cost functions when working with clinical data due to the presence of noise that is sufficiently large to obscure the global minimum, as reported in Xi et al. (2013).

### Parameter estimation workflow

The proposed parameter estimation pipeline lead to a unique estimation of the Guccione material parameters in the 8 clinical data sets analysed in this study, and with $|\Delta \varvec{u}|$ residuals (Table 2) comparable to previously reported errors (Table 3). It is important to note that the set of kinematic BCs in this study was lighter (only constraining the base, and not also the apex as in Xi et al. (2013)), thus making the task of reproducing the clinical observation more challenging.

In the proposed methodology the unique $C_1$ and $\alpha$ parameters, where the coupling occurs (Xi et al. 2013), are found while fixing two of the less intercorrelated ratios $r_f$, $r_t$, $r_{ft}$ (the third is bound by Eq. 6). Once $C_1$ and $\alpha$ are found, the ratios can be uniquely found (as reported in Xi et al. (2013)). The impact of a wrong initial choice of $r_f$, $r_t$, $r_{ft}$ on the estimation of $C_1$ and $\alpha$ parameters was evaluated in the sensitivity study (Appendix 6) and was found to be relatively low.

An important remark in the methodology is the existence of challenges associated with the convergence of nonlinear mechanics solvers in incompressible applications (Land et al. 2015b). The lack of convergence can often obstruct the calculation of the geometry CF residual for the whole $C_1$ range of interest under the known $\alpha$. Knowing that the set of coupled parameters that lead to very similar minimum costs draw a line in the $\alpha$-$C_1$ log-scale space (Xi et al. 2013) allows the problem to be reformulated as finding the parameters of this exponential line.

### Model assumptions

There are a series of model assumptions which, although not affecting the contribution of the proposed pipeline to parameter identifiability, may have an impact on the parameters found.

One important factor determining diastolic filling is the residual active tension (AT), which is known to be present in diastole (Bermejo et al. 2013; Xi et al. 2013). In this study the parameters are estimated from late diastolic instants and the inclusion of an earlier frame suggested the presence of remaining AT as detailed in Appendix 7. End-diastolic events, where the AT can be assumed to be limited and its contribution to the work estimation negligible, are the most suitable observations. Following this approach, any remaining AT at end diastole leads to an apparent increased myocardial stiffness (Asner et al. 2015), specifically in the fibre direction (Xi et al. 2013).

The most important element in the proposed methodology was revealed to be the choice of the reference frame (see Appendix 6), in concordance with previous studies (Xi et al. 2013). The reference geometry directly affects the observed myocardial stiffness as it dictates the measured strain under a given cavity pressure. In this study the LV geometry at the lowest pressure frame was chosen to describe this geometry following a popular approach (Asner et al. 2015; Gao et al. 2015; Land et al. 2012; Nikou et al. 2016; Wang et al. 2009) in order to simplify the workflow. Inclusion of a reference frame estimation (Krishnamurthy et al. 2013) can possibly enhance the parameter estimation in future applications.

Assumptions are also made regarding the definition of the myocardial microstructure and material. Conforming to the majority of FE studies in the field of cardiac mechanics, the myocardium is assumed to be incompressible although capillary and coronary flow are known to locally violate this assumption (Ashikaga et al. 2008; Yin et al. 1996). This assumption affects both the simulated deformation fields from the mechanics solver, as well as the novel energy-based CF, where deformation due to cardiac perfusion (increment of volume during diastole) is assimilated to contribute to the tissue strain energy. Also in the definition of myocardial microstructure, the inclusion of a more realistic fibre field might improve the accuracy in the estimation of the projected strain components (Eq. 3) and thus that of the energy-based CF (Eq. 18). Nevertheless, results in the sensitivity study (Appendix 6) suggest that the impact of this assumption is very small, in accordance to previous studies (Land et al. 2015a).

A central assumption in the modelling approach followed here is myocardial material homogeneity, which by reducing model complexity facilitates the parameter estimation procedure. However, this currently restricts the application of the proposed method to disease where this assumption is valid. Although rendering our method suitable for cardiac disease with localized stiffness alterations -such as myocardial infarction- is within our future plans, our workflow is readily suitable for applications on disease, such as dilated cardiomyopathy, diffuse fibrosis or heart failure with normal ejection fraction (HFnEF), where tissue properties are expected to be more homogeneous.

One last set of assumptions are needed to define the BCs of the model. First, a homogeneous pressure load is assumed to act on the endocardial boundary during ventricular filling. This is a reasonable simplification based on the reported cavity pressure variations in the literature (de Vecchi et al. 2014), and is commonly taken for computational efficiency (Gao et al. 2015; Hadjicharalambous et al. 2015; Mojsejenko et al. 2015; Nikou et al. 2016; Wang et al. 2013). However the impact of the RV, atria and pericardium on achieving more physiological deformations is known (Belenkie et al. 2001; Tyberg and Smith 1990; Williams and Frenneaux 2006), and thus the use of more advanced mechanical models (Fritz et al. 2014) is anticipated to improve model fidelity and therefore parameter estimation accuracy. We would also expect that more realistic BCs will enable the model to better reproduce the recorded myocardial deformation, thus leading to smaller residual $|\Delta \varvec{u}|$—and this should be especially beneficial in 3 of our cases (PC3, PC4 & HC, see Table 2).

### Estimated parameters in vivo

Few studies have reported passive material properties for multiple patients. The identified parameters for the 8 cases in this study (Table 2) are within the reported range in the literature (Table 3). In our results, $C_1$ falls within a range of 800–5300 Pa for human HF patients, as opposed to higher values obtained when $\alpha$ was held fixed. The $\alpha$ values fitted here span from 5 to 66 and lie within the literature range, while the higher values corresponding to HF patients are significantly lower to the ones previously reported when fixing $C_1$ at 2000 Pa (Table 3).

**Fig. 7:**
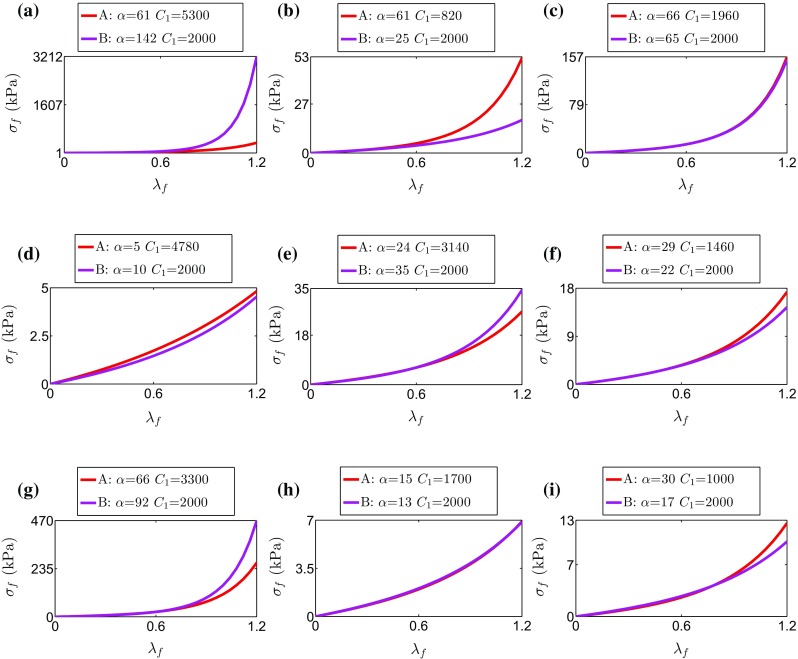
Cauchy stress vs stretch curves for an idealized 1-D extension along the fibre direction (up to 120% stretch) of an incompressible cube with the Guccione law using the material parameters estimated with the proposed methodology (A: energy-based & $|\Delta \varvec{u}|$ CFs) and a previous one where one of the parameters was assigned a certain value (B: $C_1$ fixed at 2000 Pa & $|\Delta \varvec{u}|$ CF as in Xi et al. (2013))

In our results we found that in four out of seven patient data sets (PC1, PC3, PC5, PC7) both identified parameters were higher than those of the healthy data set, in two cases (PC2, PC6) only the $\alpha$ parameter was increased while in one case (PC4) $C_1$ was increased instead. The sample of 7 HF subjects already reveals functional differences by the linear and exponential components of the material law, and we can speculate that this may have links with the aetiology of the disease. While the general trend is for the HF patient data sets processed to have higher stiffness than the healthy case in agreement with previous clinical studies (Wang et al. 2013), further cases need to be investigated to confirm this observation.

### Cardiac Mechanics Application

Computational models are increasingly used in clinical applications. Inferring material properties from these models offers three potential applications with each benefiting from uniquely constrained parameters. Firstly, material parameters may provide a more sensitive descriptor of patient pathology (Lamata et al. 2016). To test the utility of this application first requires a method for inferring constitutive parameters from clinical data. Assuming that these parameters reflect some underlying material property, and hence reflect the patients pathology, then the inferred parameter values should be unique and independent of the fitting method.

Secondly, the use of biophysical models, that are constrained by physical laws, increases the capacity of models to predict outside of the data used to constrain them. This is particularly important for patient-specific models that could be used to predict response to treatments from pre procedure data. The use of non-unique parameters will make these predictions dependent on the parameter inference method, increasing the uncertainty in the model predictions.

Finally, models can be used for predicting values of interest that can not easily be measured, including material stress, regional work and local mechanical efficiency. We have shown that an alternative approach for a unique estimation of parameters, by fixing $C_1$, leads to significant discrepancy in model predictions (see Fig. 7).

## Conclusions

A novel and clinically tractable pipeline for passive myocardial material estimation is proposed, which manages to decouple the material constitutive law parameters and guarantee reliable material estimation. This is an important step towards the use of myocardial stiffness as a reliable tool for the understanding of cardiac pathophysiology and the development of biomechanically relevant biomarkers. This work highlights the central role of CFs in the identifiability of material parameters.

## Sensitivity analysis

This section tests the performance of the novel energy-based CF in the presence of data quality issues or arbitrary modelling assumptions that are necessarily made in clinical models. For this purpose we use the in silico workbench with access to ground truth parameter values, and then perform a sensitivity analysis. We focus on the impact in the estimation of the $\alpha$ parameter (ground truth is 30), since it is provided by the novel energy-based CF.

### Methods

#### Sensitivity to pressure offset errors

Artefacts in the pressure data were studied introducing an offset, which can be caused by calibration errors and are hypothesized to be the main source of material parameter errors (see the large impact reported in Xi et al. (2014)). The effect of offset pressure values is investigated by shifting all pressure data points by +/−10% of the mean pressure $\bar{p}$ (corresponding to a 75 Pa increase/decrease in the pressure values).

#### Sensitivity to strain data quality

The quality of the strain field in the imaging data can affect the accuracy of the estimated $\alpha$, as the energy-based CF relies on the integration of strains in two diastolic timepoints (Eq. 18). To assess the anticipated error in parameter estimation from strain data noise, we inserted white noise to the strain tensor at the gauss points used for the integration of strain energy density function in Eq. 14. The noise was independently applied to each strain component in fibre coordinates, and corresponded to a standard deviation of 5, 10 and 20% of the mean strain $\bar{E}_{ij}$, which varied from −0.15 to 0.10 in the employed diastolic frames (corresponding to increments 29 and 30 of the original simulation).

#### Sensitivity to modelling assumptions

Two modelling assumptions are challenged, the choice of the reference configuration and the mathematical description of the myocardial microarchitecture (fibre field).

The reference frame, which represents the geometrical configuration at which the myocardium is unloaded and the developing stresses and strains are zero, is difficult to calculate. In the clinical application of the parameter estimation pipeline, the reference frame is estimated by taking an observed geometry at the beginning of diastole, similar to previous approaches (Hadjicharalambous et al. 2015; Mojsejenko et al. 2015; Wang et al. 2013). To estimate the effect of an inaccurate reference configuration on the parameter solution, later frames (frames 1 to 5, where frame 0 is the original reference frame) were used as reference configurations in the mechanical model.

The cardiac microarchitecture is approximated by rule-based mathematical models (Bayer et al. 2012; Nielsen et al. 1991) with $-60^{\circ }$ to $+60^{\circ }$ angle variation. To assess the effect of this choice of fibre microarchitecture, a set of extreme vertical fibres was selected (fibre angles varying from $-90^{\circ }$ to $+90^{\circ }$).

#### Effect of fixed parameter values

Throughout the parameter estimation procedure fixed values for the exponential stiffness parameter ratios were assumed. To evaluate the effect of this arbitrary choice, two different stiffness ratio combinations were used: one pertaining to a highly anisotropic material ($r_{f}=0.85$, $r_{ft}=0.10$, $r_{t}=0.05$) and one to an isotropic material ($r_{f}=0.34$, $r_{ft}=0.33$, $r_{t}=0.33$).

### Results and discussion

**Table 5:** Sensitivity analysis results with respect to the identified $\alpha$ parameter

Data/model modification	$\varvec{\alpha _{sol}}$
Pressure	
+10% $\bar{p}$ offset	22
−10% $\bar{p}$ offset	40
$\varvec{E}$ noise	
STD 5% $\bar{E}_{ij}$	28
STD 10% $\bar{E}_{ij}$	26
STD 20% $\bar{E}_{ij}$	37
Ref. Fr.	
1	26
2	22
5	3
Fibres	
−/+ 90 $^o$	29
$r_{f}$-$r_{ft}$-$r_{t}$	
0.85-0.1-0.05	29
0.34-0.33-0.33	28

The ground truth value for $\alpha$ is 30

**Table 6:** Results of the estimation of $\alpha$ with an energy cost function extended to consider the last 3 frames of diastolic filling ($\alpha _{\text {3fr.}}$), instead of the 2 of the original formulation (referred to here as $\alpha _{\text {2fr.}}$)

Case	Synth	PC1	PC2	PC3	PC4	PC5	PC6	PC7	HC
$\alpha _{\text {3fr.}}$	30	78	79	52	1	$-^{\mathrm{a}}$	41	77	17
$\alpha _{\text {3fr.}} - \alpha _{\text {2fr.}}$	0	17	18	12	$-$4	$-^{\mathrm{a}}$	12	11	2

$^{\mathrm{a}}$ PC5 had data inconsistent with passive inflation, see text for further details

Results (see Table 5) demonstrate a satisfactory robustness of the approach to possible sources of disagreement between model and data. The largest effect on parameter estimation accuracy derives from an unsuitable choice of reference configuration (choice of frame 5, $\alpha _{\mathrm{sol}}=3$). The offset in pressure recordings has the second largest impact on the parameter estimation, with smaller parameters found with larger pressure values and vice-versa. The anticipated effect of the quality of the strain field on the estimated parameter values is also confirmed. The microstructural representation in the model seemed to have a negligible effect on the identified values. The stiffness anisotropy ratios did not bias the estimation of $\alpha$ (despite the pronounced anisotropy vs isotropy scenarios tested) but had a larger effect on the estimation of $C_1$, which was 2100 Pa for the highly anisotropic assumption and 620 Pa for the isotropic one. In both cases the resulting $|\Delta \varvec{u}|$ residual of the forward simulations with the identified parameters was increased from 3 $10^{-9}~\hbox {mm}$ for the ground truth parameters to 1.15 and 1.07 mm, respectively.

## The effect of using additional frames in the energy-based CF on $\alpha$

In our analysis the two last frames of diastole were used as they provide larger strain signals with lowest probability for presence of residual AT. Alternatively, additional late diastolic frames can be included in the analysis, leading to a modified functional using the ratios of the paired combinations of the last 3 diastolic frames $f_{3DF}$ (Eq. 20):

20 $$\begin{aligned} f_{3DF}= \left| \frac{{W_{\mathrm{ext}}}_1}{{W_{\mathrm{ext}}}_2}-\frac{{W_{\mathrm{int}}}_1}{{W_{\mathrm{int}}}_2} +\frac{{W_{\mathrm{ext}}}_2}{{W_{\mathrm{ext}}}_3}-\frac{{W_{\mathrm{int}}}_2}{{W_{\mathrm{int}}}_3} +\frac{{W_{\mathrm{ext}}}_1}{{W_{\mathrm{ext}}}_3}-\frac{{W_{\mathrm{int}}}_1}{{W_{\mathrm{int}}}_3}\right| \end{aligned}$$

Results in Table 6 show that the change in $\alpha$ is generally small (up to a 25% except for a 40% in PC6). In most of the cases the additional earlier frame led to a larger value in $\alpha$, suggesting the presence of residual AT. The data of the additional earlier diastolic frame in PC5 indicated a decrease in strain energy, despite pressure increasing, which is incompatible with underlying model assumptions and results in a negative $\alpha$ value.

## An alternative approach for unique parameter estimation

In this work a novel CF was proposed that can isolate $\alpha$, allowing for $C_1$ to be uniquely determined from previous geometrical CF. The proposed energy CF relies on strain fields derived directly from the images, and thus on the availability of high quality data and robust image registration tools (Shi et al. 2012). A similar approach can be followed by exploiting the fact that the cavity pressure that inflates the myocardium to a given volume scales linearly with the parameter $C_1$. This leads to the definition of an alternative pressure based CF, which relies on the good quality of the pressure data and their synchronization to the volume trace:

21 $$\begin{aligned} f_p=\left| \frac{p_{\mathrm{dat1}}}{p_{\mathrm{dat2}}} - \frac{p_{\mathrm{sim1}}}{p_{\mathrm{sim2}}}\right| \, \end{aligned}$$

**Table 7:** Parameter estimation results from the pressure-based CF (pCF), compared to the energy-based CF (eCF)

Case	$\alpha ^{pCF}$	${C_1}^{pCF}$	$\alpha ^{pCF}-\alpha ^{eCF}$	${C_1}^{pCF}-{C_1}^{eCF}$
Synth	30	1000	0	0
PC1	18	21630	$-$43	16330
PC2	6	8335	$-$55	7515
PC3	${>}120^{\mathrm{a}}$	<885$^{\mathrm{a}}$	>54$^{\mathrm{a}}$	<$-$1075$^{\mathrm{a}}$
PC4	50	240	45	$-$4540
PC5	60	1070	36	$-$2070
PC6	130	275	101	$-$1185
PC7	>400$^{\mathrm{a}}$	<210$^{\mathrm{a}}$	>334$^{\mathrm{a}}$	<$-$3090$^{\mathrm{a}}$
HC	50	310	45	$-$1390

Note that ${C_1}$ here is obtained in both cases by the L$^2$ displacement norm

$^{\mathrm{a}}$ Results in PC3 and PC7 are bounded since the passive inflation simulation for larger $\alpha$ values did not converge

where $p_{\mathrm{dat1}}, p_{\mathrm{dat2}}$ are the measured cavity pressures at two timepoints in diastole and $p_{\mathrm{sim1}} , p_{\mathrm{sim2}}$ are the esti at the two last diastolic frames (DF2, DF1 are again the end-diastolic and pre-end-diastolic frames from the MRI sequence, respectively) and ${p_{\mathrm{sim}}}^{DF_1}$, ${p_{\mathrm{sim}}}^{DF_2}$ are the estimated cavity pressures at the respective timepoints, which are required to inflate the reference mesh to the measured cavity volumes. For consistency with the energy CF analysis, these diastolic timepoints are defined as frames $\mathrm{DF}_{1}$ and $\mathrm{DF}_{2}$ corresponding to the pre-end-diastolic and end-diastolic frames of the MRI sequence (Sect. 2.3.3). As $p_{\mathrm{sim}}$ scales with $C_1$, the ratio $\frac{p_{\mathrm{sim1}}}{p_{\mathrm{sim2}}}$ is independent of $C_1$ and therefore $f_p$ depends only on $\alpha$ (for fixed anisotropy ratios). Note that the pressure CF can only be evaluated through forward simulations, while in contrast the energy CF can be evaluated directly from the clinical data.

To evaluate the pressure form of the cost function we find the $\alpha$ parameter for the 8 cases of this study. For this purpose, parameter sweeps over $\alpha$ for an arbitrary fixed $C_1$ at 2000 Pa were performed. For each $\alpha$ the myocardium was inflated to an elevated cavity pressure (3 times the end-diastolic pressure) in 90 increments. The values $p_{\mathrm{sim1}}$ and $p_{\mathrm{sim2}}$ were found by linear interpolation between the two simulation increments with volumes that bound the cavity volume from the data. Following estimation of $\alpha$ from Eq. 21, $C_1$ was estimated from the already fitted exponential curves to the minimum L$^2$ displacements norm CF residual (as explained in Sect. 2.4) and the results are presented in Table 7.

Despite the fact that both the energy CF and the pressure CF do find the correct unique set of parameters in an in-silico test, parameters show large differences depending on the CF when working with real data (see Table 7). This is a similar finding as when working with the collection of geometrical CFs, and reflects the incongruity between data and model accentuated by the different origins and types of data errors. The assumption that these patients, who share a common pathology, have similar material properties would suggest that for the available data the energy-based CF provides a more robust estimate of model parameters. Nevertheless, there is no ground truth methodology available for the evaluation of the performance of the different CFs. Further research in data acquisition and analysis, on model fidelity, and on alternative in-vivo strategies may improve the agreement between the different CFs and lead to the true material parameters of the beating myocardium.

## Application of the energy-based CF to the reduced Holzapfel–Ogden material model

The method can be extended to a variety of exponential parameter laws where scaling parameters are used in one or more terms. An example that suits this description is the Holzapfel–Ogden (H-O) law (Holzapfel and Ogden 2009):

22 $$\begin{aligned} \begin{aligned} \varPsi&=\frac{a}{2b} \left[ \mathrm {e}^{b(I_1-3)}-1\right] +\frac{a_f}{2 b_f} \left[ \mathrm {e}^{b_f(I_{4f}-1)^2}-1\right] \\&\quad + \frac{a_s}{2 b_s} \left[ \mathrm {e}^{b_s(I_{4s}-1)^2}-1 \right] + \frac{a_{fs}}{2 b_{fs}} \left[ \mathrm {e}^{b_{fs} I_{8fs}^2}-1\right] \end{aligned} \end{aligned}$$

where:

23 $$\begin{aligned} \begin{aligned}&I_1=tr(\varvec{C}) \, \,&\, \, I_{4f}=\varvec{f}_0 \cdot (\varvec{C}\varvec{f}_0) \\&I_{4s}=\varvec{s}_0 \cdot (\varvec{C}\varvec{s}_0) \, \,&\, \, I_{8fs}=\varvec{f}_0 \cdot (\varvec{C}\varvec{s}_0) \end{aligned} \end{aligned}$$

and $\varvec{f}_0,~\varvec{s}_0$ the basis vectors aligned with the local fibre and sheet directions, respectively. Following the approach by Hadjicharalambous et al. (2015) we will use the reduced version of this law where the parameters associated with the sheet and fibre–sheet response are set to zero ($a_s,a_{fs}=0$). This provides an equivalence in the number of unknown parameters between the Guccione and reduced H–O (both have four parameters). Aiming to address the main direction of coupling in the reduced H–O, which is expected to occur between the scaling parameters and those inside the exponential function (pairs: *a*-*b*, $a_f$-$b_f$), we use the parameter ratios $\gamma =\frac{a}{a_f}$ and $\delta =\frac{b}{b_f}$ to reformulate the reduced H–O relationship as:

24 $$\begin{aligned} \varPsi= & {} \frac{\gamma a_f}{2 \delta b_f} \left[ \mathrm {e}^{\delta b_f (I_1-3)}-1\right] +\frac{a_f}{2 b_f} \left[ \mathrm {e}^{b_f(I_{4f}- 1)^2}-1\right] ,\nonumber \\ \end{aligned}$$

25 $$\begin{aligned} \varPsi= & {} \frac{a_f}{2 b_f} \left\{ \frac{\gamma }{\delta } \left[ \mathrm {e}^{\delta b_f (I_1-3)}-1\right] + \left[ \mathrm {e}^{b_f(I_{4f}- 1)^2}-1\right] \right\} .\nonumber \\ \end{aligned}$$

From Eq. 25 the energy-based CF for the reduced H-O law can be expressed as :

26 $$\begin{aligned} f_{rH-O}= & {} \left| \frac{W_{ext1}}{W_{ext2}}- \frac{ \frac{a_f}{2 b_f} \left\{ \frac{\gamma }{\delta } \left[ \mathrm {e}^{\delta b_f ({I_{11}}-3)}-1\right] +\left[ \mathrm {e}^{b_f({I_{4f1}}- 1)^2}-1\right] \right\} }{\frac{a_f}{2 b_f} \left\{ \frac{\gamma }{\delta } \left[ \mathrm {e}^{\delta b_f ({I_{12}}-3)}-1\right] + \left[ \mathrm {e}^{b_f({I_{4f2}}- 1)^2}-1\right] \right\} } \right| \nonumber \\ \end{aligned}$$

27 $$\begin{aligned} f_{rH-O}= & {} \left| \frac{W_{ext1}}{W_{ext2}}- \frac{ \frac{\gamma }{\delta } \left[ \mathrm {e}^{\delta b_f ({I_{11}}-3)}-1\right] + \left[ \mathrm {e}^{b_f({I_{4f1}}- 1)^2}-1\right] }{\frac{\gamma }{\delta } \left[ \mathrm {e}^{\delta b_f ({I_{12}}-3)}-1\right] + \left[ \mathrm {e}^{b_f({I_{4f2}}- 1)^2}-1\right] } \right| \nonumber \\ \end{aligned}$$

and by fixing the two parameter ratios $\gamma$, $\delta$-in analogy to our application for the Guccione law $f_{rH-O}$ is only a function of $b_f$ if $I_1$, $I_{4f}$ are estimated directly from the data deformation field. In Eq. 26, 27 $W_{ext1}, I_{11}$ and $I_{4f1}$ and $W_{ext2}, I_{12}$ and $I_{4f2}$ denote the external work, $I_1$ and $I_{4f}$ invariants at two timepoints which correspond to diastolic frames $DF_1$ and $DF_2$ respectively. Following estimation of $b_f$, the scaling parameter $a_f$-in analogy to the formulation for the Guccione law can be estimated from $|\Delta \varvec{u}|$.

To demonstrate the ability of this CF to isolate $b_f$ we have performed an in silico analysis, where the synthetic LV presented in Sect. 2.1.1 with the same cardiac microstructure (Sect. 2.2.2) was assigned a material behaviour described by Eq. 25 with $a_f=10,000~\hbox {Pa}$, $\gamma =2.5$, $b_f=5$, $\delta =1$ as in Hadjicharalambous et al. (2015), and was passively inflated to 1500 Pa in 15 increments. In analogy to the in silico test for the Guccione law, the ‘frames’ corresponding to the last two increments of the simulation were chosen as $DF_1$ and $DF_2$ and the energy-based CF presented a unique minimum at $b_f=5$, thus identifying the ground truth value.

## Footnotes

1
www.cmiss.org/cmgui.

2
www.itksnap.org.

3
www.cheart.co.uk.
